# Gene expression prediction based on neighbour connection neural network utilizing gene interaction graphs

**DOI:** 10.1371/journal.pone.0281286

**Published:** 2023-02-06

**Authors:** Xuanyu Li, Xuan Zhang, Wenduo He, Deliang Bu, Sanguo Zhang

**Affiliations:** 1 School of Mathematical Sciences, University of Chinese Academy of Sciences, Beijing, China; 2 Key Laboratory of Big Data Mining and Knowledge Management, Chinese Academy of Sciences, Beijing, China; 3 Institute for Network Sciences and Cyberspace (INSC), Tsinghua University, Beijing, China; 4 Zhongguancun Laboratory, Beijing, China; 5 School of Statistics, Capital University of Economics and Business, Beijing, China; Hanyang University, KOREA, REPUBLIC OF

## Abstract

Having observed that gene expressions have a correlation, the Library of Integrated Network-based Cell-Signature program selects 1000 landmark genes to predict the remaining gene expression value. Further works have improved the prediction result by using deep learning models. However, these models ignore the latent structure of genes, limiting the accuracy of the experimental results. We therefore propose a novel neural network named Neighbour Connection Neural Network(NCNN) to utilize the gene interaction graph information. Comparing to the popular GCN model, our model incorperates the graph information in a better manner. We validate our model under two different settings and show that our model promotes prediction accuracy comparing to the other models.

## Introduction

Gene expression data, which describe the process of converting DNA materials into functional products [1], has been an important tool for medical diagnosis and gaining insights into complex disease [2, 3]. With the advance in DNA microarray [4] and RNA-seq technologies [5, 6], the cellular response can be studied through thousands of expression data under a wide variety of conditions such as diseases, genetic mutations and intake of medicines and drugs. The corresponding study is called gene expression profiling.

Although lots of gene expression data have been collected and deposited [7, 8], whole genome profiling is still too expensive for broad use since it requires the collection of data with a large number of genes through various conditions. For example, The initial phase of the CMap project produced only 564 genome-wide gene expression profiles [9]. One of the solutions to reduce the expense of whole genome profiling is to utilize the high correlation among different genes [10] and select a group of genes to represent overall genome expression. Researchers from the LINCS program performed principal components analysis(PCA) and found that 1,000 carefully chosen genes(named landmark genes) were sufficient to recover 80% of the information in the whole genome [11]. Then they developed the L1000 Luminex bead technology to measure the expression profiles of these 1000 genes at a much lower cost. Lots of literature have been proposed then based on this cost-effective strategy [12, 13].

Despite the low cost of the L1000 program, one of the natural questions is how to infer other genes, named target genes, based on these landmark genes. The original paper proposed by the LINCS program adopts simple linear regression. Although classic and computationally efficient, linear regression can not capture the nonlinear relationship between landmark genes and target genes. With the development of deep learning methods, Li et al. [10] proposed a full connection neural netword-based method D-GEX and achieved better results than linear regression in both DNA-microarray and RNA sequencing data.

Although D-GEX performs much better than traditional methods, it may be further improved. D-GEX uses full connection neural network model which implicitly assuames that landmark genes are interchangeable. In other words, the landmark gene expression data can be fed into the full connection neural network in any order without affecting the final result. The motivation of this paper comes from considering whether this implicit assumption holds for the gene expression data. As shown in many biology studies [14, 15], the genes have an inherent structure, at the same time, cells can coordinate the regulation of many genes at once. Thus, the D-GEX model neglects the latent structure of the landmark genes, and it is beneficial to incorporate exterior information which gives the structure of genes into the deep learning method. The gene interaction graph, which depicts such coordination by giving functional biological interaction between two genes is a perfect candidate. In the gene interaction graph, nodes represent genes, and edges represent the functional biological interaction between two genes. There have been many gene interaction graphs constructed from different molecular levels(Szklarczyk et al. [16]; Warde-Farley et al. [17];): protein-protein interaction, transcription factors, and gene co-expression are the common material to construct gene interaction graph. Another aspect is that in the deep learning literature, the processing of graph data has recently drawn a major interest [18]. Any neural network working on the graph data can be categorized as a graph neural network(GNN). In particular, graph convolutional network(GCN) has been a predominant approach [19] among the graph neural networks.

In this paper, we will briefly introduce the classical graph convolutional network structure and then compare the GCN with our method which tackles some deficiencies of the original structure and successfully improves the prediction accuracy. Our main contribution is to propose a novel neural network architecture to utilize the gene interaction graph. We give some explanation of why our method is better than the popular graph covolutional network model and D-GEX. We further run experiments on two different datasets to validate that our model improves the prediction accuracy with fewer paramters.

## Related works

### Deep neural network for gene expression prediction

D-GEX is a full connection neural network using approximately 1000 landmark genes as input and approximately 9000 target genes as output. Due to computational reasons, the target genes were separated into two parts, and D-GEX was independently trained in two parts.

D-GEX was trained with MSE(mean squared error) as a loss function based on GEO expression data which we will further introduce in detail in the next section. The hyperbolic tangent (TANH) function is chosen as the activation function and other training techniques including dropout, and momentum acceleration are also applied. Candidate models include neural networks with 1 to 3 hidden layers each with 3000, 6000 and 9000 hidden sizes validated based on The GTEx expression data and MAE(Mean absolute error) is chosen as the model evaluation metric.

Recently, several deep neural networks have been used for gene expression prediction. Wang et al [20] use Conditional Generative Adversarial Networks to model the conditional probability of target genes given landmark genes. Kunc and Kléma [21] substitutes the hyperbolic tangent function with transformative adaptive activation functions to improve the prediction accuracy. Wang et al [22] use a recurrent neural network called L-GEPM to model the non-linear features of the landmark genes. However, just like D-GEX, these methods do not take the structure of the target genes into consideration. Our work mitigatea this issue by utilizing gene interaction graphs.

### Graph neural networks using external information

There have been works done to use the gene interaction graph for gene expression prediction, yet their tasks somewhat differ from ours.

Not restricting the genes to the 943 landmark genes and 9520 target genes, Dutil et al. [23] use GNN to do one gene expression prediction, which is termed a Single Gene Inference task. The output in their task is only one gene expression and its input genes are selected by picking the closest genes to the output gene in the gene interaction graph, which is different from the one in D-GEX [10]. Because the latter restricts input and output to the landmark genes and target genes predefined in the literature.

A line of research has been done following Dutil’s work. Bertin et al. [24] find that the effect of incorporating randomly-generated networks to improve the prediction accuracy can be almost as well as that of biological networks. This finding suggests that with respect to the gene expression data, the biological networks may not be a good prior knowledge. However, Crawford et al. [25] further investigate that after removing the low-degree genes, the biological network brings better effects than random graphs. Trebacz et al. [26] uses the ontology embedding of genes to improve Single Gene Inference task accuracy.

To the best of our knowledge, there have not been any studies to incorporate the gene interaction graph to improve prediction accuracy in D-GEX. So in this paper, we will incorporate the gene interaction graph as prior information into the deep learning model for better prediction accuracy.

## Method

### Data

Two datasets were used named as GEO dataset [10] and GTEx dataset [27]which were also included in the D-GEX paper. After a similar pre-processing protocol with D-GEX, the GEO dataset consists of 111009 gene expression profiles from the Affymetrix microarray platform and The GTEx dataset consists of 2921 gene expression profiles from the Illumina RNA-Seq platform. Both datasets consist of 943 landmark genes as input and 9520 target genes as output.

In the following section, we will conduct experiments on these two datasets and compare the performance of D-GEX, GCN, linear regression and our method. We conduct the task in two settings:

1. For the microarray platform, we split the GEO dataset into 80% for training, 10% for validation and 10% for testing.
2. For the RNA-Seq platform, we directly split the GTEx dataset into 80% for training, 10% for validation and 10% for testing.

The first setting in our paper is similar to D-GEX to illustrate that the proposed method generates better results in the microarray platform. However, the second setting is significantly different from D-GEX which directly tests GTEx dataset with the neural network trained with GEO data. It can be seen from the original paper of D-GEX and the results in our paper that the prediction error is much higher if the train set and test set are from different platforms. To further state that our proposed methods perform better results on different platforms and on a different number of data, we directly split GTEx dataset as a train set and a test set.

Besides, we additionally take the gene interaction graph data as prior biological information to construct the model.

In this paper, we use STRING database [16], which is available online at https://string-db.org. The String database saves all publicly available sources of protein-protein interaction graphs. The edges in the STRING database have seven types: three prediction edges based on genomics context information (see below), and one type respectively for co-expression, text-mining, previously curated pathway and protein-complex knowledge (‘databases’). In this paper, we use the co-expression protein interaction graph. With the gene-protein mapping rules, we can further obtain the gene interaction graph as prior information. The original co-expression graph contains 2945888 edges between 18520 gene nodes. We obtain the sub-graph by restricting the 943 landmark genes and the corresponding edges, which is needed in our task.

### Neighbour connection neural network

The effectiveness of machine learning methods largely depends on the nice representation of the data. A nice representation of the data should be concise but informative to better serve the machine learning task. Traditionally, representation learning is based on human effort which is called feature engineering [28]. Recently, the deep learning method dominates the traditional machine learning method in many areas. The success of deep learning is mainly due to its ability to automatically learn the representation of the original data. So the key factor in promoting a new successful neural network architecture is to better learn the data representation.

For the machine learning task of the image data, it is very important to learn a good representation of the image. Proposed by Lecun et al. [29], the Convolutional Neural Networks have achieved outstanding performance in machine learning tasks in image data [30].

By capturing the local pattern of the image, CNN learns good representation. To be general, the image data can be viewed as a special graph that consists of grids of nodes and RGB node attributes. So it is desired to generalize the local pattern extraction technique used in CNN to the graph data to gain better data representation.

However, the main difference between image data and graph data is that the pixel has a regular structure. Any pixel is adjacent to the 8 pixels surrounding it, so it is possible to use weight sharing for every local connection feature map. On the other hand, the graph does not possess this property because the degree between nodes is usually different. So we proposed a new method named Neighbour Connection Neural Network(NCNN), which intuitively captures the local pattern by only connecting the neighbour of a node.

We denote the graph as G=(V,E), where V is the set of *d* nodes and E is the edge set. *A* is a *d* × *d* adjacency matrix, with its entries *A*_*ij*_(binary or weighted) denoting the strength of connectivity between node *i* and node *j*. In this paper, we consider a gene interaction graph where nodes represent genes and edges represent the biological association between genes. The attribute on each node is a uni-variate gene expression level, so we assume that the attribute on a node is one-dimensional, and denote the node attributes on the graph as *X* = (*X*_1_, *X*_2_, …, *X*_*d*_)^*T*^.

In this paper, the original gene interaction graph we use has 943 nodes and 26545 edges with weighted attributes. In this case, *d* = 943, |E|=26545, and *A*_*ij*_ denotes the biological association between node *i* and node *j*. The edge number is so large that connecting the neighbour will not result in local pattern extraction. So in order to capture the local pattern of the graph, we can firstly drop the weak signal edge to control the sparsity of the network.

A natural way to sparsify the gene network is by setting a threshold to filter the weak connection between nodes. Formally, we set:

$$\begin{array}{c} A_{i,j}=\{\begin{array}{cc} 1 & A_{i,j}>\delta \\ 0 & A_{i,j}<\delta . \end{array} \end{array}$$ (1)

The threshold is selected through running experiments by picking the threshold performing best in the prediction accuracy. With cross-validation from candidate set {120, 121, …, 150}, we pick 137 as the threshold *δ*. We use $N\left(i\right)=\left\{j:A_{ij}=1\right\}$ to denote the neighbours of node *i*.

Given a gene expression *X* = (*X*_1_, …, *X*_*d*_)^*T*^ as input, we embed it in a sparse graph G=(V,E). Just like the multi-layer feed-forward neural network, the neighbour connection neural network has one input layer, many hidden layers and one output layer. However, the forward propagation is done on the graph G. Starting from the input layer, we denote the node attribute as layer 0 node representation *H*^(0)^ = *X* ∈ *R*^*d*×1^. Assuming there are *L* hidden layers, the node representation of layer *l* is denoted as $H^{\left(l\right)}\in R^{d\times h_{l}}$ which is different from the D-GEX. In D-GEX, the hidden representation is a vector. But in our method, the hidden feature is learned on the node of the graph and in the form of the matrix. ($H_{i,m}^{\left(l\right)}$ means the hidden feature *m* on the node *i*). We denote the hidden feature *m* of all the nodes on the graph as $H_{\cdot ,m}^{\left(l\right)}$.

In CNN, the feature is learned by locally connecting the adjacent pixel and the pixel itself to the next layer. So in our method, the representation on a node is propagated with the node neighbours.

The Neighbour Connection forward propagation goes as:

$$\begin{array}{c} H_{\cdot ,m}^{\left(l+1\right)}=\sigma \left(\Sigma_{k=1}^{h_{l}}W^{m,k,l}H_{\cdot ,k}^{\left(l\right)}+b^{m,k,l}\right)\;m=1,\ldots h_{l+1}, \end{array}$$ (2)

where *W*^*m*, *k*, *l*^ ∈ *R*^*d*×*d*^ is the sparse weight matrix connecting neighbours of a node along with the node itself in the hidden layer *l* to the node in the hidden layer *l* + 1. *m* stands for the *m*th dimension of the hidden feature vector in the hidden layer *l* + 1 and *k* stands for the *k*th dimension of the hidden feature vector in the hidden layer *l*. For every pair of the one dimension of hidden feature in hidden layer *l* and the one dimension of hidden feature in hidden layer *l* + 1, there is a weight matrix *W*^*m*, *k*, *l*^ connecting them. The entries $W_{i,j}^{m,k,l}$ in *W*^*m*, *k*, *l*^ are set to 0 with respect to the sparse adjacency matrix *A*.

1. If *A*_*i*, *j*_ = 0, $W_{i,j}^{m,k,l}=0$.
2. If *A*_*i*, *j*_ > 0, $W_{i,j}^{m,k,l}$ is a trainable parameter.

If *A*_*i*, *j*_ = 0, it means that node *i* is not a neighbour of node *j*, so there is no connection between them, then $W_{i,j}^{m,k,l}=0$. If *A*_*i*, *j*_ > 0, it means that node *i* is the neighbour of node *j* and the representation on the node *j* in the layer *l* + 1 will incorporate this node information, thus $W_{i,j}^{m,k,l}$ is a trainable parameter. The hidden feature in layer *l* + 1 is then learned by first linearly suming the hidden feature in layer *l* scaled by different weight matrix and a bias term *b*^*m*, *k*, *l*^, then transformed the linear sum with a non-linear activation function *σ*.

This formula can also be written in the node-wise form:

$$\begin{array}{c} \begin{array}{cc} H_{i,m}^{\left(l+1\right)} & =\sigma \left(\Sigma_{k=1}^{h_{l}}W_{i\cdot }^{m,k,l}H_{\cdot k}^{\left(l\right)}+b_{i}^{m,k,l}\right)\;i=1,\ldots d,m=1,\ldots ,h_{l+1}\\ & =\sigma \left(\Sigma_{k=1}^{h_{l}}\Sigma_{j\in N(i)\cup \{i\}}W_{ij}^{m,k,l}H_{j,k}^{\left(l\right)}+b_{i}^{m,k,l}\right)\;i=1,\ldots d,m=1,\ldots ,h_{l+1}. \end{array} \end{array}$$ (3)

**Fig 1:**
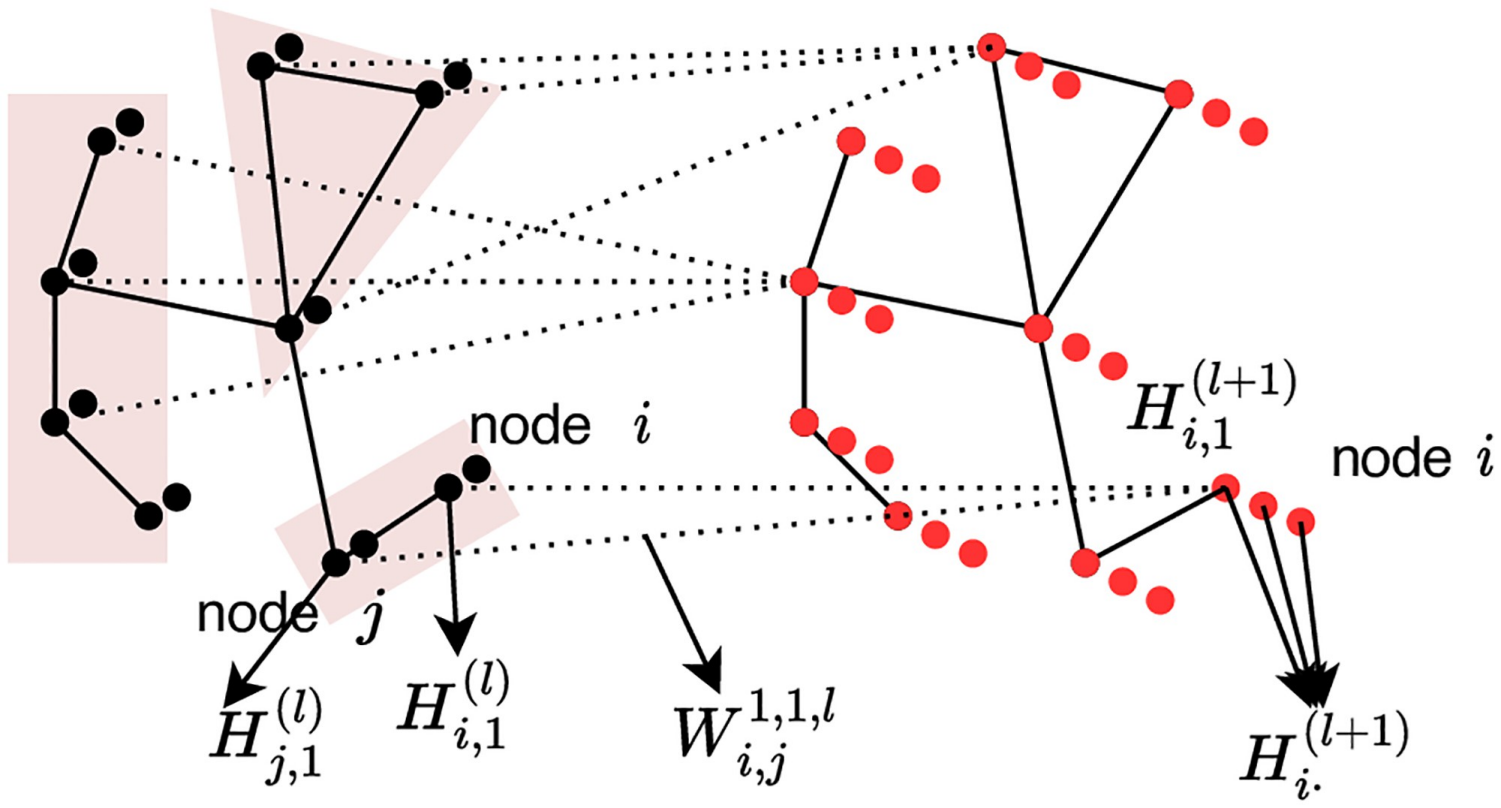
Detailed illustration of neighbour connection. This figure illustrates the Neighbour Connection of node *i* in a more detailed way. The node *i* has only one neighbour *j*. And the figure draws the linear scaling factor from the hidden feature 1 in layer *l* to the hidden feature 1 in layer *l* + 1 with respect to the node *i* and its neighbouring node *j*.

It can be seen more clearly in the node-wise form and the illustration figure that by using a sparse linear transformation matrix *W*^*j*, *k*, *l*^, the representation of node *i* in layer *l* + 1 is learned by locally connecting the representation of its neighbour N(i) along with itself in the layer *l*. Moreover, for each connection from node *j* feature *k* in layer *l* to the node *i* in the layer *l* + 1 feature *j*, there is a unique trainable parameter $W_{im}^{j,k,l}$ to scale the corresponding neighbour information. To better present our method, we illustrate the Neighbour Connection of the node *i* in Fig 1.

To fully make use of the representation learned through neighbour connection layer, we then flatten all the attributes and connect them to a full connection neural network model.

In this paper, we just consider using one layer of neighbour connection because:

1. As suggested before, the gene interaction signal is weaker than the expression level. So performing the neighbour connection too many layers will deteriorate the gene expression information. A similar problem also exists in GCN, which is called ‘over-smoothing’.
2. Practically, one layer of neighbour connection is enough to capture the local pattern and shows its significant improvement over D-GEX, so we perform one neighbour connection layer for simplicity.
3. In order to compare with full connection neural network, we set our model to have the same layer including the neighbour connection although with much fewer parameters. The D-GEX has three layers in total, and if we use more than one neighbour connection layer there will be only one layer for full connection. Although the local feature is extracted, it is hard to use only one hidden layer to learn the global feature and get a good prediction.

Because we only use one neighbour connection layer and the input representation on each node is a one-dimensional gene expression level. We rewrite the formula of NCNN when *l* = 0 and *h*_0_ = 1.

In this case, *h*_0_ in Eq (3) is 1, the node-wise Neighbour Connection is:

$$\begin{array}{c} H_{i,m}^{\left(1\right)}=\sigma \left(\sum_{j\in N(i)\cup \{i\}}W_{i,j}^{m,1,l}H_{j,1}^{\left(0\right)}+b_{i}^{m,1,l}\right)\;i=1,\ldots d,m=1,\ldots ,h_{1}. \end{array}$$ (4)

Then we flatten $H_{i,m}^{\left(1\right)}$ matrix into a vector *H*^(1)^:

$$\begin{array}{c} H^{\left(1\right)}={\left(H_{\cdot ,1}^{{\left(1\right)}^{\prime }},H_{\cdot ,2}^{{\left(1\right)}^{\prime }},\ldots ,H_{\cdot ,h_{1}}^{{\left(1\right)}^{\prime }}\right)}^{\prime }. \end{array}$$ (5)

The hidden feature in layer 1 is *H*^(1)^ and will be further connected to an MLP model.

**Fig 2:**
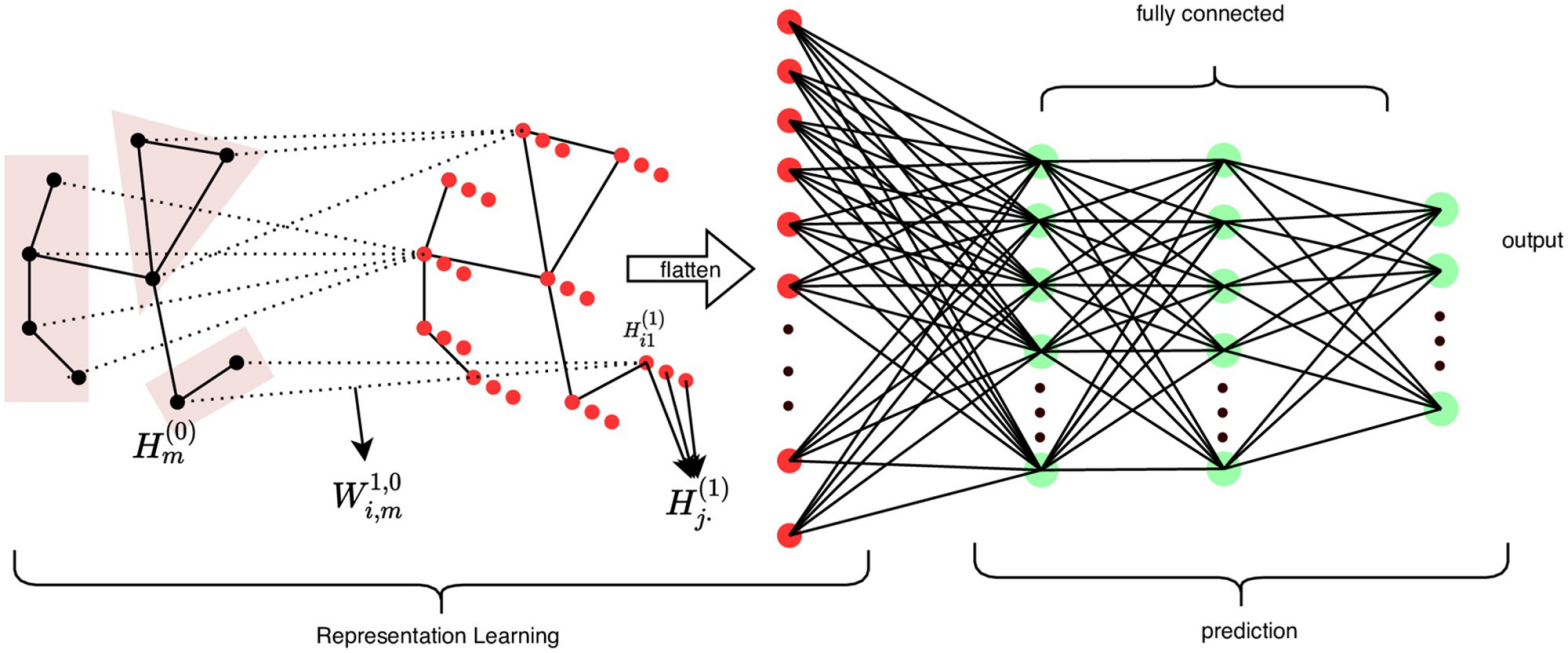
Structure illustration. The structure of the NCNN model is composed of the neighbour connection layer with a flattening operation and the full connection layer.

The structure of our NCNN model is illustrated in Fig 2.

In order to train our model better, we adopt several training techniques including:

1. Instead of using mean squared loss to train the model, *L*_1_ loss is adopted as the loss function. This is because *L*_2_ loss is vulnerable to outliers and will yield a smoothing effect [31], yet the *L*_1_ loss is roboust to outliers and can lead to accurate prediction.
2. We change the activation function in the MLP part of our model to log sigmoid function and find that it can better train the NCNN.

### Comparison of GCN

In the line of research on Single Gene Inference task [23–26], the authors use Graph Convolutional Networks(GCN) to utilize the graph information. Our method is somewhat motivated by the GCN but improves its drawbacks, so we will briefly introduce this method and illustrate the difference between the GCN and our proposed method.

There are a lot of Graph Convolutional Networks proposed [19, 32–34]. We just explain the Graph Convolutional Network proposed by Moris et al. [34]. It is one of the most widely used graph neural network architectures because of its simplicity and effectiveness.

The node representation in the layer *l* + 1 in the graph convolutional network propagates in the following way:

$$\begin{array}{c} H^{\left(l+1\right)}=\sigma \left({\tilde{D}}^{-\frac{1}{2}}\tilde{A}{\tilde{D}}^{-\frac{1}{2}}H^{\left(l\right)}W^{\left(l,1\right)}+H^{\left(l\right)}W^{\left(l,2\right)}\right). \end{array}$$ (6)

Here $\tilde{A}=A+I$ is the adjacency matrix of the graph with self-connection, which can incorporate the node representation itself in the propagation. $\tilde{D}$ is a diagonal matrix with ${\tilde{D}}_{ii}=\sum_{j=1}^{d}{\tilde{A}}_{ij}$. *σ* is the activation function like Hyperbolic tangent function, Relu function or Sigmoid function. $W^{\left(l,1\right)},W^{\left(l,2\right)}\in R^{h_{l}\times h_{l+1}}$(*h*_*l*_ and *h*_*l*+ 1_ are respectively the dimension of node representation in the layer *l* and layer *l* + 1.) are two linear transformation matrix whose entries are all trainable parameters.

The propagation can be understood more clearly in the node-wise form:

$$\begin{array}{c} H_{i,\cdot }^{\left(l+1\right)}=\sigma \left(\sum_{j\in N(i)}\frac{A_{ij}}{\sqrt{{\tilde{D}}_{ii}{\tilde{D}}_{jj}}}H_{j,\cdot }^{\left(l\right)}W^{\left(l,1\right)}+\frac{1}{{\tilde{D}}_{i}}H_{i,\cdot }^{\left(l\right)}W^{\left(l,1\right)}+H_{i,\cdot }^{\left(l\right)}W^{\left(l,2\right)}\right). \end{array}$$ (7)

From this formula, we know that the graph convolutional neural network propagates the representation on a node by combining the neighbours’ representation scaled with the edge attribute *A*_*i*, *j*_, then the weight matrix *W*^*l*+1^ transforms the linear-combined *d*_*l*_ dimensional feature into the *d*_*l*+1_ dimensional feature.

Just like the Eq (4), we consider the case when *l* = 0 and *h*_0_ = 1, the GCN formulation is:

$$\begin{array}{c} H_{i,j}^{\left(1\right)}=\sigma \left(\sum_{j\in N(i)}\frac{A_{ij}}{\sqrt{{\tilde{D}}_{ii}{\tilde{D}}_{jj}}}H_{j}^{\left(0\right)}W_{j}^{\left(0\right)}+\frac{1}{{\tilde{D}}_{i}}H_{i}^{\left(0\right)}W_{j}^{\left(0\right)}\right)i=1,\ldots d,j=1,\ldots ,h_{1}. \end{array}$$ (8)

We can see from Eqs (4) and (8) that the NCNN uses more parameters than the GCN: every node in NCNN has a different linear transformation matrix $W_{i,j}^{j,0}$. Yet in GCN, all the nodes share the same matrix *W*^0^.

However, the graph convolutional neural network is designed to learn the representation of a graph. In the setting where GCN has nice performance like social network [19], recommendation system [35] and quantum chemistry [36], the edge is a key component of the graph. In other words, the edge is a strong signal in these settings. So by simply scaling node attributes with edge attributes and combining them, the representation is learned well and efficiently.

In our task, gene expression data plays a much more important role while the edge is only side information. It is not a strong connection relationship signal [16]. Linearly combining adjacent representation scaled by edge attribute will deteriorate the important signal of the original node input, because the node feature information is deteriorated by scaling with the less important edge feature.

## Results and discussion

### GEO dataset

Firstly, we compare the results of different models on setting 1 including D-GEX, NCNN, GCN and LR(Linear Regression). To show the supremacy of our proposed NCNN model over others, we conduct the experiments under approximately the same hidden nodes and hidden layers with different sets of node numbers and layer numbers.

Precisely, as mentioned above, we only use one layer of neighbour connection in order to compare with the D-GEX. After one layer of neighbour connection, the representation is fed into an MLP model just the same as D-GEX.

To have approximately the same hidden nodes and hidden layers, say *H* hidden nodes and *L* hidden layers, we perform one layer of neighbour connection, whose *h*_1_ is set as $\left[\frac{H}{d}\right]$. So that after neighbour connection and flattening, the length of *H*^(1)^ is $d*[\frac{H}{d}]$, approximately equaling the hidden nodes *H* in the D-GEX model. Then *H*^(1)^ is fed into an MLP model with *H* hidden nodes and *L* − 1 hidden layers. The GCN model setting is similar to the NCNN.

It is very clear in Table 1 that our proposed model has better prediction performance than the other models. To begin with, the linear regression model has the worst performance because the model only captures the linear relationship between the input and output variables. While the deep learning models including the D-GEX, NCNN and GCN model the non-linearity between the variables and can better make predictions.

**Table 1:** MAE comparison between D-GEX and NCNN model in the prediction of GEO data when varying the number of hidden layers and hidden size.

method	hidden size			
	hidden layers	3000	6000	9000
D-GEX	1	0.3421 ± 0.0858	0.3337 ± 0.0869	0.3300 ± 0.874
	2	0.3377 ± 0.0854	0.3280 ± 0.0869	0.3224 ± 0.0879
	3	0.3362 ± 0.0850	0.3252 ± 0.0868	**0.3204 ± 0.0879**
NCNN	1	0.3570 ± 0.0830	0.3493 ± 0.0843	0.3432 ± 0.0849
	2	0.3186 ± 0.0843	0.3067 ± 0.0860	**0.3025 ± 0.0871**
	3	0.3175 ± 0.0837	0.3067 ± 0.0854	0.3032 ± 0.0860
GCN	1	0.3587±0.0876	0.3532±0.0889	0.3512±0.0892
	2	0.3504±0.0855	0.3487±0.0864	**0.3473±0.0872**
	3	0.3502±0.0842	0.3539±0.0839	0.3523±0.0844
LR	0.3780 ± 0.0848			

Among the three different deep learning methods, the best result of D-GEX is achieved with 3 hidden layers and 9000 nodes, and the corresponding mean average error is 0.3204. GCN achieves its best results with 2 hidden layers and 9000 hidden nodes, and the corresponding mean average error is 0.3473, which is worse than the original D-GEX. Our proposed model NCNN performs best with 2 hidden layers and 9000 nodes, with the mean average error 0.3023. NCNN has shown its supremacy over other methods not only in the prediction accuracy, but in the number of parameters as well. Just as we have analyzed, although the GCN model utilizes the gene interaction information, the way it incorporates this side information will deteriorate the original gene expression signal. At the same time, NCNN uses a distinct parameter to scale the neighbour information so that it better uses the gene interaction graph. It only uses 2 hidden layers including one neighbour connection layer and one full connection layer to achieve the best results.

**Fig 3:**
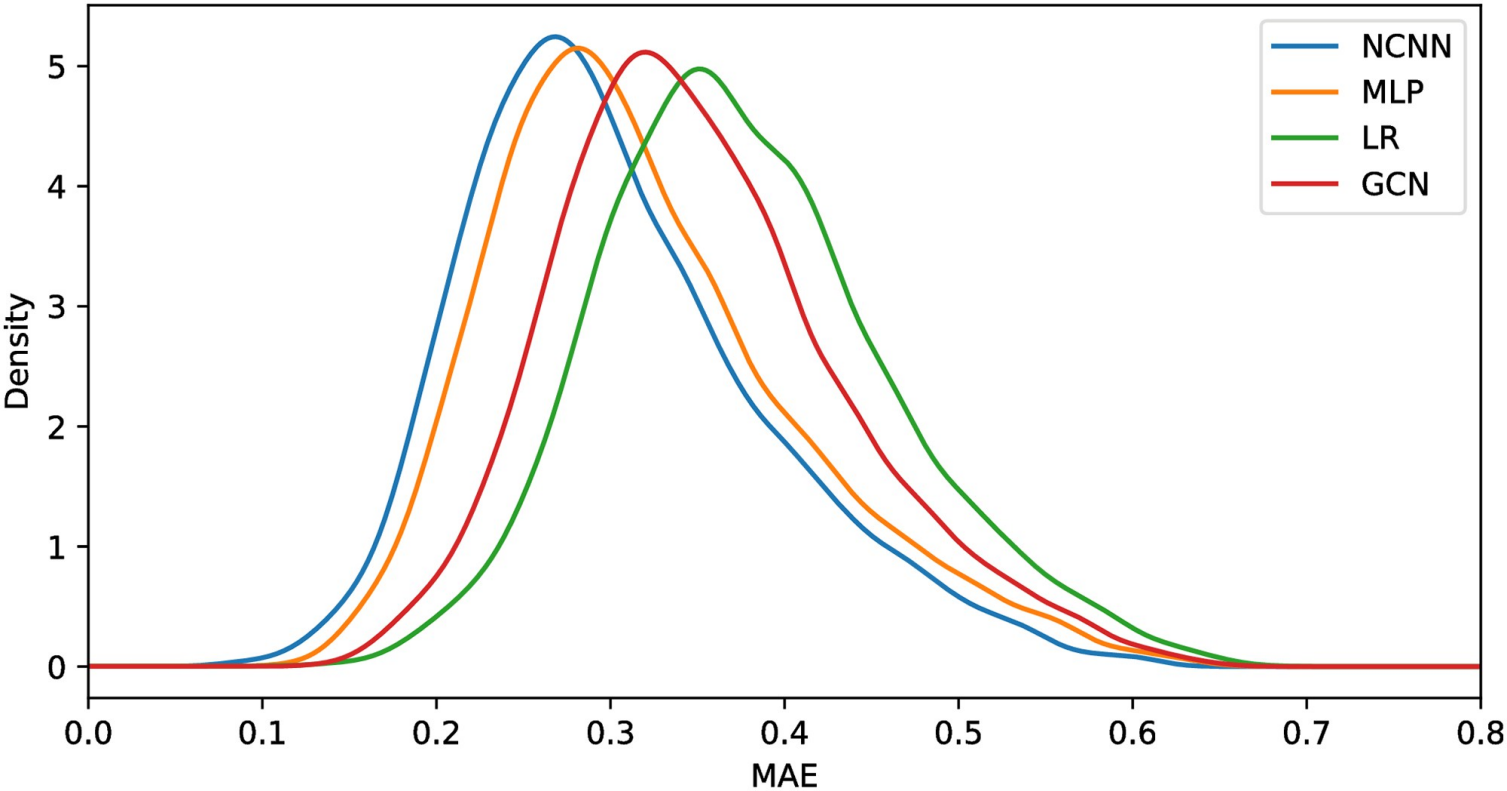
Density plot of different models with the best structure.

Fig 3 shows the density plot of the mean average error per gene of four models. This figure shows that the peak of the density function of NCNN has smaller MAE than the comparing methods. And the density function of NCNN is bigger than the comparing methods when MAE is small and smaller than the comparing methods when MAE is large. Showing that it has precise prediction on more genes and vague prediction on less genes.

**Fig 4:**
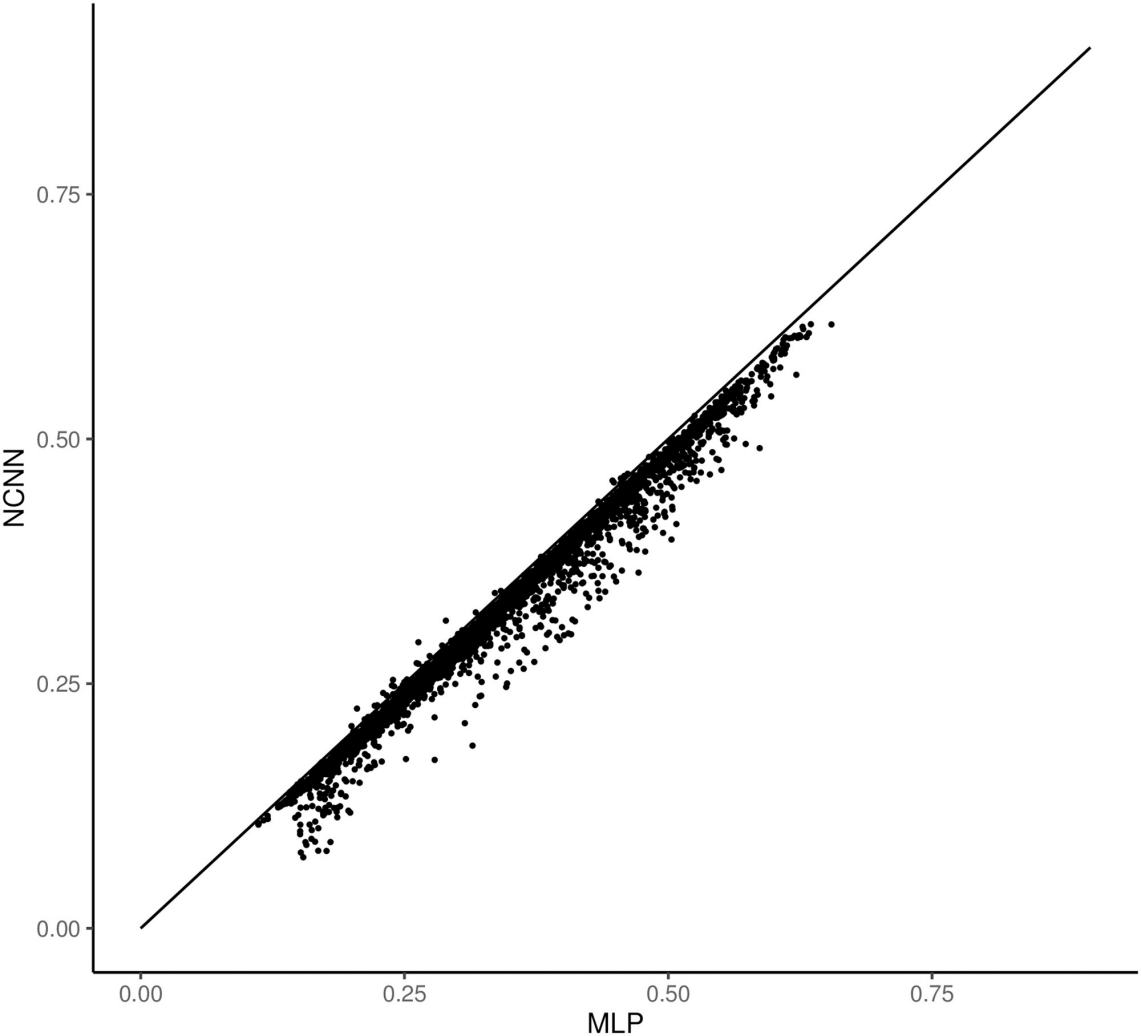
The comparison of mean average error of predictions on each gene in GEO dataset. The y value is the mean average error from NCNN model on each target gene; the x value is the mean average error from MLP model on each target gene. Each dot below the diagonal means that NCNN has lower prediction error on this gene than the comparing method.

**Fig 5:**
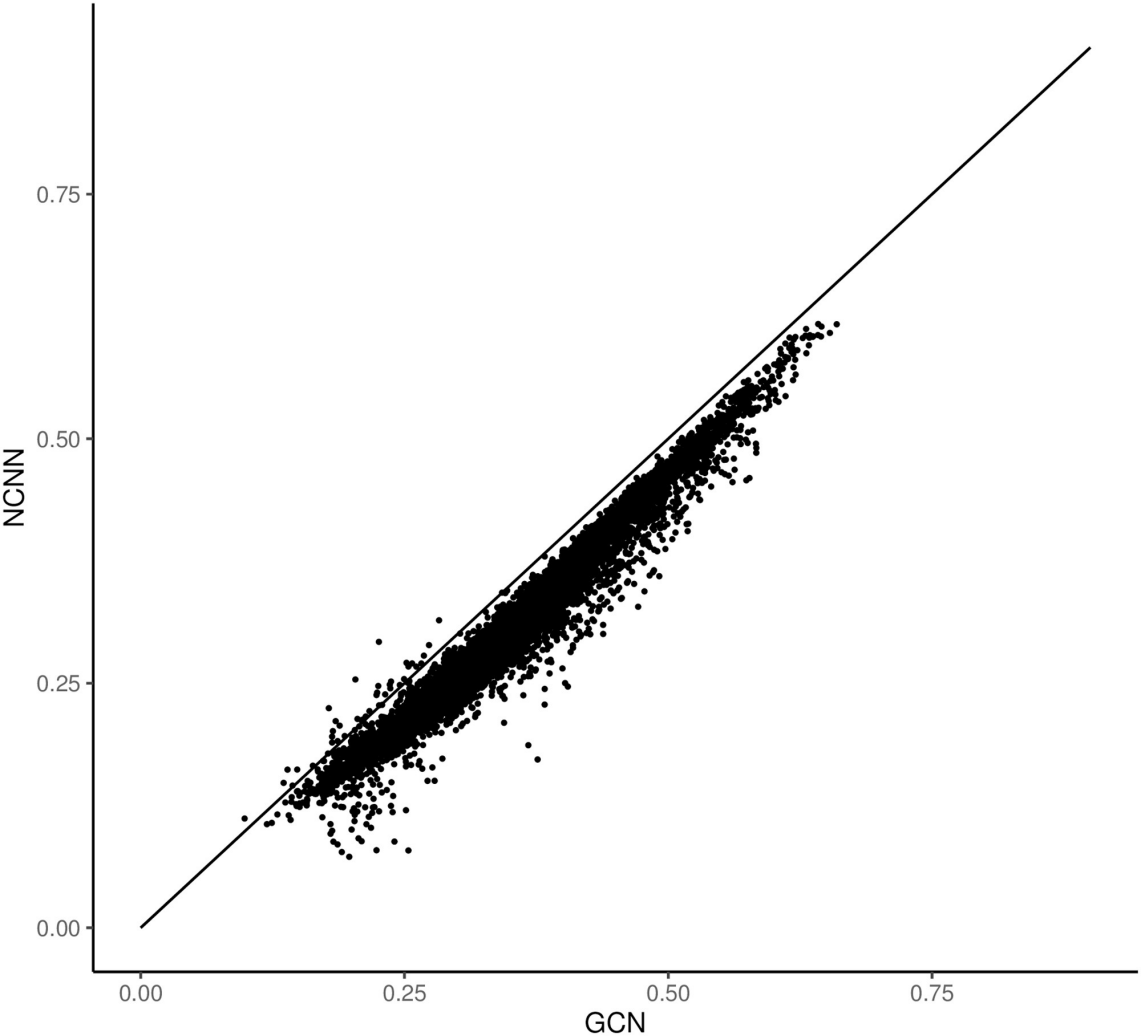
The comparison of mean average error of predictions on each gene in GEO dataset. The y value is the mean average error from NCNN model on each target gene; the x value is the mean average error from GCN model on each target gene. Each dot below the diagonal means that NCNN has lower prediction error on this gene than the comparing method.

Figs 4 and 5 show the comparative error figure of the two models on each gene. It is clear that the NCNN outperforms other models significantly at the gene-wise level. It makes better predictions on 99.66% genes than MLP, and on 99.57% genes than GCN.

We analyze the complexity of linear transformation parameters in three models as shown in Table 2. In the full connection layer used in D-GEX, if the input length is *d* and the output length is *n*, then the number of parameters is *dn*. In the NCNN, we embed the *d* dimensional input into a graph with *d* nodes and perform neighbour connection. The parameters we use are $\sum_{i=1}^{d}\left|N\left(i\right)\right|\left[\frac{n}{d}\right]$, normally we use sparse connection graph, so $\sum_{i=1}^{d}\left|N\left(i\right)\right|=2|E|$ is in the complexity of *O*(*d*), then the overall parameters we use has the complexity of *O*(*n*), this is less than the full connection parameters *dn*. The GCN also embeds the *d* dimensional input into a graph with *d* nodes and performs convolution. It only uses 2[*n*/*d*] = *O*(*n*/*d*) parameters, which is the least among the three methods.

**Table 2:** The parameter complexity of different models and the number of parameters they actually use.

model	paramters complexity	number of parameters
D-GEX	*dn*	8568000
NCNN	*O*(*n*)	95787
GCN	*O*(*n*/*d*)	18

### GTEx dataset

We then compare the results of different models on setting 2 including D-GEX, NCNN, GCN and LR(Linear Regression) in Table 3. Just like setting 1, we conduct the experiments under approximately the same hidden nodes and hidden layers with different sets of node numbers and layer numbers.

**Table 3:** MAE comparison between D-GEX and NCNN model in the prediction of GTEx data when varying the number of hidden layers and hidden size.

method	hidden size			
	hidden layers	3000	6000	9000
D-GEX	1	0.2160± 0.0749	0.2165± 0.0762	0.2181± 0.0770
	2	0.2148± 0.0741	**0.2118± 0.0758**	0.2132± 0.0770
	3	0.2181± 0.0744	0.2133± 0.0748	0.2176± 0.0770
NCNN	1	0.2194±0.0748	0.2177± 0.0749	0.2159± 0.0749
	2	0.2138± 0.0733	0.2115± 0.0736	0.2113± 0.0740
	3	0.2146± 0.0735	0.2077± 0.0729	**0.2072± 0.0730**
GCN	1	0.2232±0.0773	0.2254±0.0778	**0.2166±0.0770**
	2	0.2370±0.0769	0.2245±0.0759	0.2216±0.0756
	3	0.2380±0.0784	0.2374±0.0785	0.2473±0.0795
LR	0.2672 ± 0.1018			

Just like the case in setting 1, the deep learning model shows its supremacy over the traditional linear regression model.

Among the three different deep learning methods, the best result of D-GEX is achieved with 2 hidden layers and 6000 nodes, with the corresponding mean average error 0.2084. GCN achieves its best results with 1 hidden layer and 6000 hidden nodes, with the corresponding mean average error 0.2166, which is still worse than the original D-GEX. Our proposed model NCNN performs best with 3 hidden layers and 9000 nodes, with a mean average error 0.2043.

The best performance structure of the three deep learning models in setting 2 differs from that in setting 1. The D-GEX and GCN models achieve the best performance under fewer nodes and layers. This is maybe due to the low sample amount of the dataset, which leads to over-fitting. As we have mentioned, the MLP model neglects the order of the input variables and is easy to over-fit. The GCN model takes into account its order, though it uses few parameters and deteriorates the neighbour information by scaling with edge attributes. Our proposed NCNN not only takes the order of variables into account, but has distinct parameters for the neighbour of each node on the graph. So it is not so easy to get overfitted. In setting 2 where the sample is few, it achieves the best results using more layers, indicating that it learns a better representation that can be used to make better predictions.

The best structure result indicates that the GEO dataset and GTEx dataset are two distinct datasets. However, our proposed NCNN model still outperforms other models, showing its supremacy on different platforms.

**Fig 6:**
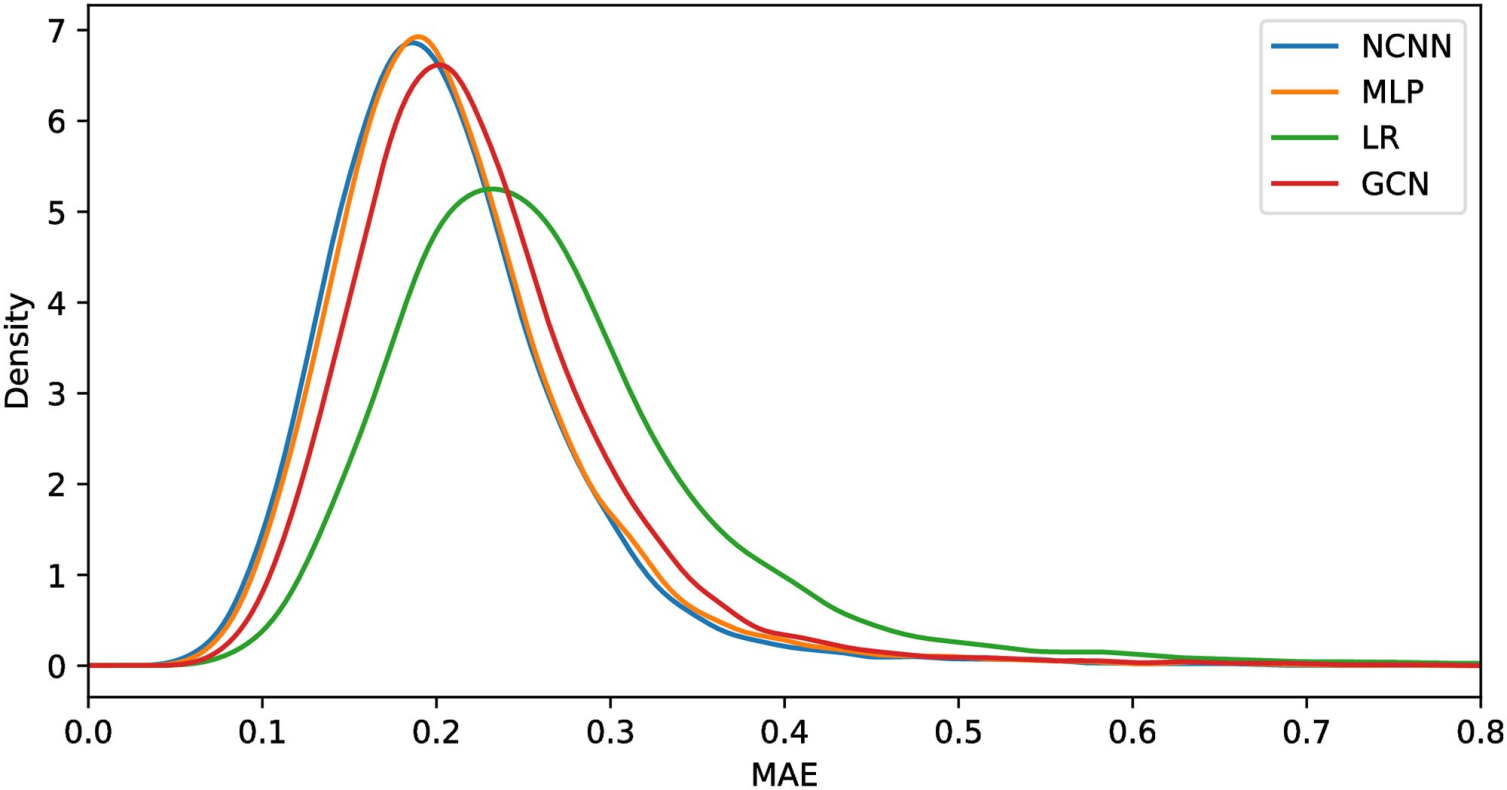
Density plot of different models with the best structure.

We also analyze the mean average error on each gene in GTEx dataset. Fig 6 shows the density plot of the mean average error per gene of four models. This figure shows that the peak of the density function of NCNN still has smaller MAE than the comparing methods. However, the difference between the MLP and NCNN is not as big as that in the GEO datasets. This is mainly because the GTEx data is generated from a totally different gene platform from the GEO data. The density function of NCNN is bigger than the comparing methods when MAE is small and smaller than the comparing methods when MAE is large. Showing that it has precise prediction on more genes and vague prediction on less genes.

**Fig 7:**
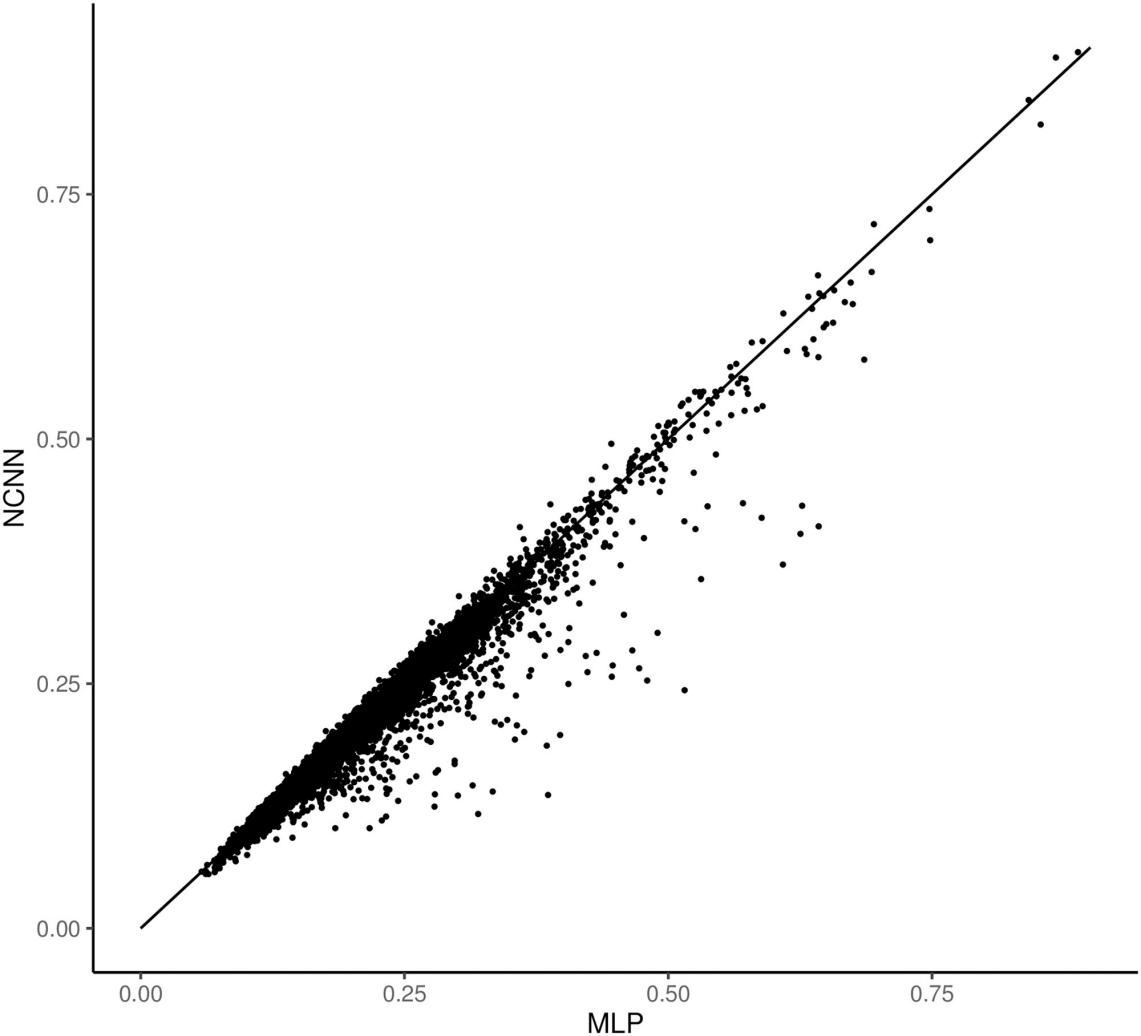
The comparison of mean average error of predictions on each gene in GTEx dataset. The y value is the mean average error from NCNN model on each target gene; the x value is the mean average error from MLP model on each target gene. Each dot below the diagonal means that NCNN has lower prediction error on this gene than the comparing method.

**Fig 8:**
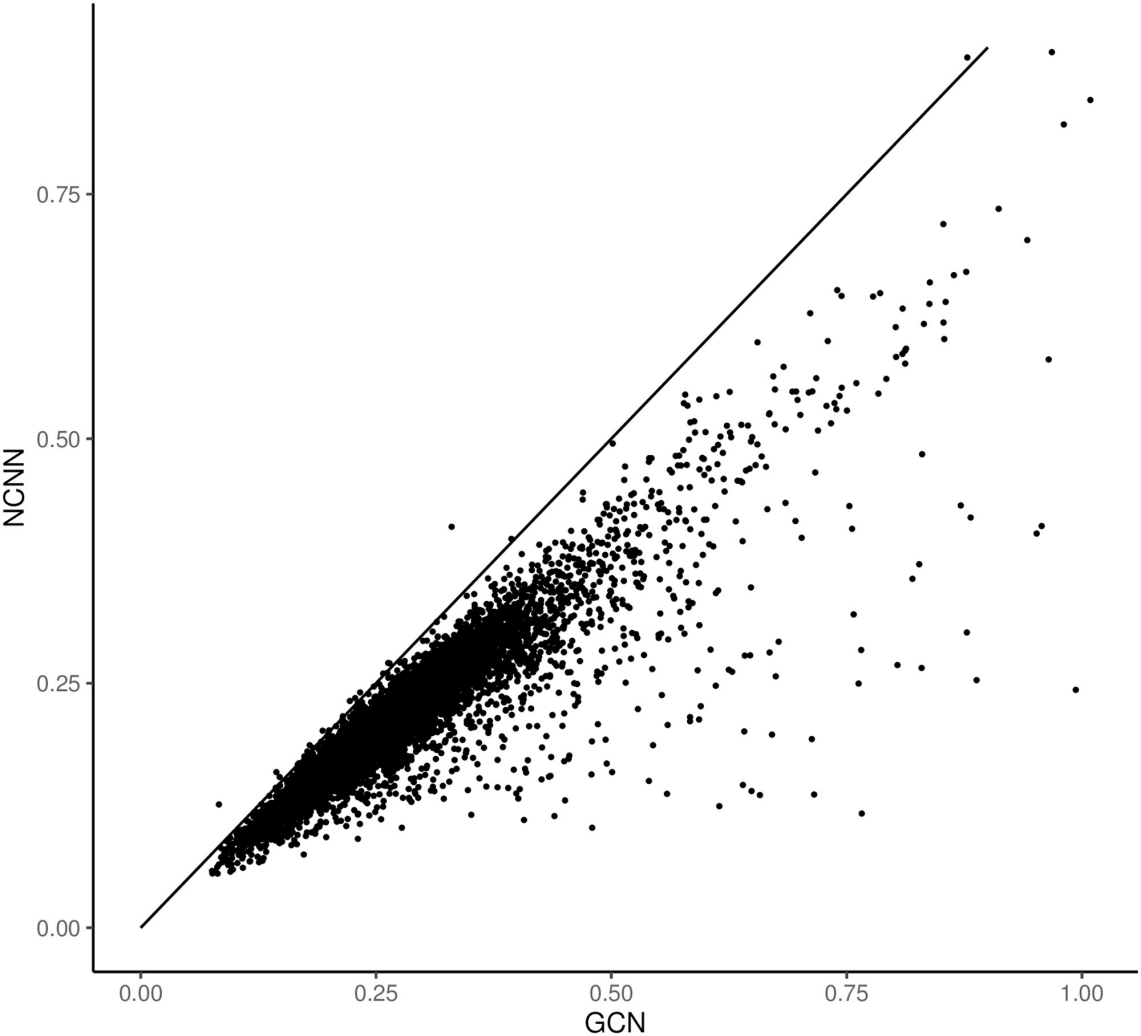
The comparison of mean average error of predictions on each gene in GTEx dataset. The y value is the mean average error from NCNN model on each target gene; the x value is the mean average error from GCN model on each target gene. Each dot below the diagonal means that NCNN has lower prediction error on this gene than the comparing method.

Figs 7 and 8 show the comparative error figure of the two models on each gene. The NCNN still outperforms other models at the gene-wise level. It makes better predictions on 59.64% genes than MLP, and on 81.51% genes than GCN. The superiority is not as big as that in the GEO data as well. And we attribute this to the distinction between the two datasets collection platform.

## Conclusion and future work

According to the special structure of genes, this paper proposes a novel neural network called the Neighbour Connection Neural Network utilizing gene graph interaction information. Our method not only theoretically learns the good representation of the gene expression value by having a reasonable inductive bias on the gene data, but outperforms the comparing methods which do not consider the structure of genes like MLP or which incorporate the structure information in a bad manner like GCN.

We validate the performance of our model on two datasets, the GEO dataset and GTEx dataset. To achieve the best results on the GEO dataset, the proposed NCNN has much fewer parameter than the D-GEX. The NCNN only uses 1.18% parameters of that in D-GEX in the first weight matrix and one less hidden layer, yet improves the mean average error by 5.6%. On the gene-wise level, our model has lower mean average error on 99.66% of the total genes than D-GEX. Although our model NCNN has more parameters than the GCN, the mean average error of NCNN is 12.8% lower than the GCN. On the gene-wise level, our model has lower mean average error on 99.57% of the total genes than GCN.

To achieve the best results on the GTEx dataset, the proposed NCNN has more parameters than the D-GEX. The NCNN only uses 1.18% parameters of that in D-GEX in the first weight matrix, but uses one more hidden layer and more hidden nodes. NCNN improves the mean average error of D-GEX by 5.6%. On the gene-wise level, NCNN has lower mean average error on 60.23% of the total genes than D-GEX. Comparing to the GCN model, the mean average error of NCNN is 4.33% lower. On the gene-wise level, NCNN has lower mean average error on 99.78% of the total genes than GCN.

This indicates that gene interaction values may have an inner structure and this information can be exploited to help the deep learning model learn better representation.

The proposed model NCNN has the advantage of better representation learning and fewer paramters than the full connection, but it also has the drawback that in practical implementation the running time is rather long due to the lack of an efficient algorithm. Besides, we only do preliminary research on how to utilize the gene graph information with our model. There are a number of future works to be done to improve our model:

- There are many types of gene networks. In this paper, we only use the String network. Thus, integrating different types of graphs is a further direction.
- We only perform one neighbour connection layer for simplicity, although our method has fewer parameters than the full connection layer, our implementation with the Pytorch library is not efficient in the computing sense. Further research can be done to accelerate the training process.
- This paper only considers the graph structure in the input layer, yet it can be further researched whether there is a way to incorporate the topology of the output variables into the neural network for further improvement.
- Our method generalizes the local pattern capture of CNN, while there are other components of CNN like pooling or sub-sampling not generalized which can be further researched.
